# Popular Cures

**Published:** 1909-10-30

**Authors:** Ashton Beck

**Affiliations:** Bromyard, Worcester


### POPULAR CURES.

Dear Sir,—I have just read with interest the article upon some popular cures. Could you find space for the following case, which appears to me to be worth recording.

My gardener's brother-in-law was reported to be dangerously ill. He had pneumonia. I asked how he was one day, and was told that he had recovered, but that this was not due to his club doctor, who was no good. He was cured by the application of sheep's lungs to the soles of his feet; it was described to me how the countryside was scoured for the lungs. This, combined with a bottle of brandy a day, was the treatment that put to shame our profession. Now hear the proof of the efficacy of sheep's lungs. This case appears to have been single pneumonia—this in itself is worthy of note. You have probably observed that "double pneumonia and pleurisy" is the usual and correct thing. Fortunately for science this case was exceptional, and the pneumonia was confined to one side; and it was found that the lungs applied to the foot corresponding to the affected side rapidly became putrid and stinking, whereas those applied to the other foot remained fresh and sweet.

These things happened in Wales, the land of faith and enthusiasms. Yours truly,

Ashton Beck.

Bromyard, Worcester.

